# Genomic divergence and demographic history of *Quercus aliena* populations

**DOI:** 10.1186/s12870-023-04623-y

**Published:** 2024-01-09

**Authors:** Biao Han, Boqiang Tong, Jiliang Zhang, Ziheng Bu, Lijun Zhao, Yang Xian, Dezhu Li, Xiaoman Xie

**Affiliations:** 1 Key Laboratory of State Forestry and Grassland Administration Conservation and Utilization of Warm Temperate Zone Forest and Grass Germplasm Resources, Shandong Provincial Center of Forest and Grass Germplasm Resources, Ji’nan, 250102 Shandong China; 2 School of Life Sciences, Shandong University, Qingdao, 266237 Shandong China; 3 3Germplasm Bank of Wild Species, Kunming Institute of Botany, Chinese Academy of Sciences, Kunming, 650201 Yunnan China

**Keywords:** *Quercus aliena*, Genetic divergence, Demographic history, Phylogeography

## Abstract

**Background:**

*Quercus aliena* is a major montane tree species of subtropical and temperate forests in China, with important ecological and economic value. In order to reveal the species’ population dynamics, genetic diversity, genetic structure, and association with mountain habitats during the evolutionary process, we re-sequenced the genomes of 72 *Q. aliena* individuals.

**Results:**

The whole chloroplast and nuclear genomes were used for this study. Phylogenetic analysis using the chloroplast genome dataset supported four clades of *Q. aliena*, while the nuclear dataset supported three major clades. Sex-biased dispersal had a critical role in causing discordance between the chloroplast and nuclear genomes. Population structure analysis showed two groups in *Q. aliena.* The effective population size sharply declined 1 Mya, coinciding with the Poyang Glaciation in Eastern China. Using genotype–climate association analyses, we found a positive correlation between allele frequency variation in SNPs and temperature, suggesting the species has the capacity to adapt to changing temperatures.

**Conclusion:**

Overall, this study illustrates the genetic divergence, genomic variation, and evolutionary processes behind the demographic history of *Q. aliena*.

**Supplementary Information:**

The online version contains supplementary material available at 10.1186/s12870-023-04623-y.

## Background

Phylogeography investigates how geographical barriers, climatic variation, and geological changes have affected the geographical distribution of genetic diversity that results from the ecological and evolutionary processes driving gene flow, population contraction, and population growth [1]. Phylogeography plays a significant role in the study of historical biogeography by enabling the analysis of current evolutionary processes in light of paleoclimatic occurrences in history, or by elucidating the relationships between biological diversification, geological events, and paleoclimate change [2–4].

Understanding the phylogeographical patterns within species and the ecological and evolutionary factors that caused them is one of the main goals for evolutionary biologists [4]. Historical climate changes have significantly affected present-day species distribution and genetic diversity. For example, Quaternary climate oscillations facilitated intraspecific differentiation by strengthening already-existing geographical barriers, and significantly reduced effective population sizes [5]. Therefore, the present-day distribution patterns of intraspecific genetic variation may be the result of simultaneous action and interaction between biological traits and climate history. The genetic structure of forest trees—especially the geographical component—is very important for the management and protection of their genetic resources. Additionally, phylogeography may be useful for forecasting trends in long-term population processes as well as accurately defining target entities for conservation [6, 7].

The widely distributed *Quercus aliena* Blume (Figure S1), sometimes known as the oriental white oak, is a member of the Fagaceae section *Quercus*. It can reach a height of 30 m and has fissured, grey-brown bark. The species is common in mixed mesophytic forests at elevations between 100 to 2700 m [8, 9].

As one of the most common deciduous trees, its acorns support the fundamental food chains in the forest ecosystems, in addition to having been used by humans as food for about 10,000 years. The wood of oriental white oak is also a good material for building boats, furniture, and wood flooring for houses. It is a significant forest species in Northern China, playing major ecological roles on the southern slopes of mountains. As a result, the population dynamics of *Q. aliena* significantly affect the structure and functionality of the forest ecosystem.

Previously, analyses using several markers—including microsatellite (SSR), amplified fragment length polymorphism (AFLP), and chloroplast markers—had detected the genetic divergence and diversity of *Q. aliena* [10, 11], revealing that gene flow was frequent between populations and that Quaternary glacial events had affected population expansion and migration. However, the markers used in these studies might provide insufficient genetic information to illuminate the genomic variation and complex evolutionary history of *Q. aliena*. With advances in sequencing techniques, genomic data in particular are being used to assess population genetics [12, 13]. Scientists are now concentrating on the nuclear genome, and genome-wide scans for genetic differentiation are a useful method to look into the potential mechanisms causing population divergence. Due to their maternally inherited traits, chloroplast genomes exhibit a clear geographical structure [14, 15], and are therefore useful in phylogeographical studies [16–19]. We may therefore conduct comprehensive investigations of the genetic diversity and divergence of *Q. aliena* by integrating chloroplast and nuclear genome sequences.

In this study, we re-sequenced the genomes of 72 *Q. aliena* individuals from across China. We assembled the whole chloroplast genome and called the SNPs using the reference genome of *Q. dentata.* We inferred patterns of genomic variation in *Q. aliena*. Next, we compared the genetic divergence between the chloroplast genome and nuclear genome. Finally, we evaluated the population demographic history and investigated the genotype associated with climate variation.

## Results

### Variation in chloroplast genomes of *Quercus aliena* populations

The length of the *Q. aliena* chloroplast genome ranged from 161,159 to 161,344 bp (Table S1). The alignment of the *Q. aliena* chloroplast genome, including sequences from 72 individuals, was 162,079 bp in length and contained 589 variable sites (0.36%) and 351 parsimony-informative sites (0.22%). The overall genetic diversity was 0.00044; moreover, three regions (IR, LSC, and SSC) revealed different sequence divergences, and the IR region exhibited the smallest variable sites (N:14). Within the 800 bp windows, three intergenic spacers (*trnG-trnR-atpA*, *psbM-trnD*, and *trnS-psbZ-trnG*) had the highest sequence divergence (Figure S2). We identified 161 indels in the chloroplast genomes of the 72 *Q. aliena* accessions, with most located in non-coding regions.

### Discordance relationships between nuclear and chloroplast genome

**Fig. 1:**
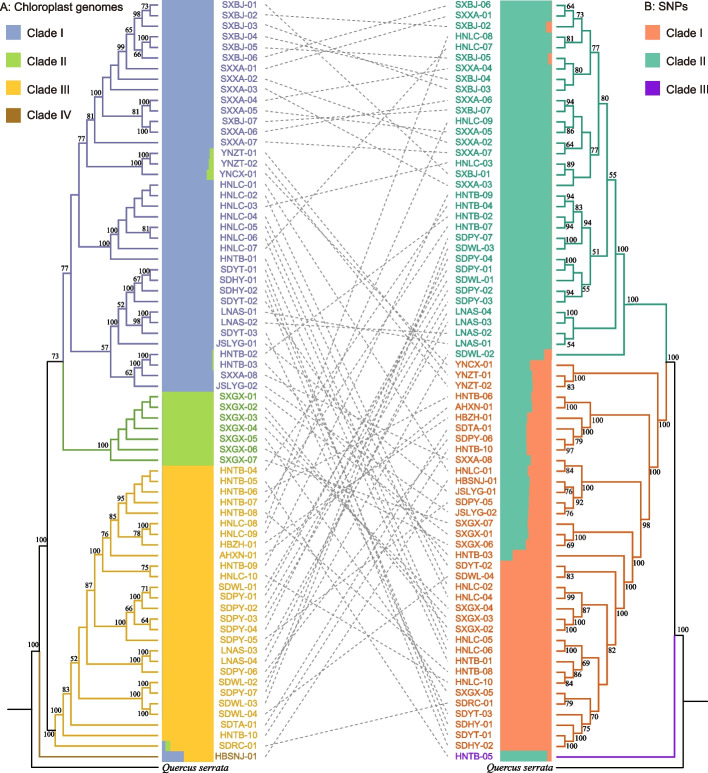
Comparison between topologies inferred from (**A**) the chloroplast genome and (**B**) nuclear SNPs. Bootstrap supports of more than 50% of ML are shown above branches. The population structures of the chloroplast genome and nuclear SNPs are shown close to the phylogenetic trees. Every vertical bar represents a single individual, and the height of each color represents the probability of assignment

The phylogenetic tree based on complete chloroplast genome sequences supported the separation of the 72 accessions into four clades (Fig. 1) with high bootstrap supporting values (Fig. 1A, and Table S1). Thirty-seven accessions belonging to 10 populations from Henan, Jiangsu, Liaoning, Shaanxi, Shandong, and Yunnan formed Clade I, including 27 haplotypes. Clade II contained 7 samples comprising one population from Guxian in Shan Xi province and was sister to Clade I, with 100% support value. Clade III included 27 accessions comprising 8 populations from Anhui, Henan, Liaoning, Hebei, and Shandong. Clade III had 19 haplotypes and was sister to clades I and II, with high support value. Clade IV only included one sample from Shennongjia in Hubei province. The accessions from the populations of HNLC, HNTB, and LNAS did not from a clade (Table S2).

The phylogenetic tree based on analysis of all the SNPs of the 72 accessions is shown in Fig. 1B. The SNP-based tree revealed three major clades—Clade I, Clade II, and Clade III, and showed that Clade I and Clade II were closely related and that both were sister to clade III, with strong bootstrap support (BS = 100). Clade I consisted of 37 accessions comprising 16 populations in Henan, Hebei, Shandong, Anhui, Jiangsu, Shaanxi, Shanxi, Hubei, and Yunnan; Clade II consisted of 37 accessions including 34 accessions comprising seven populations in Henan, Liaoning, Shandong, and Shaanxi. Clade I was sister to Clade II. Clade III only consisted of one accession, which originated from the population of Tongbai in Henan province. Both the chloroplast genome tree and nuclear SNP tree were not consistent with the populations’ geographical distributions (Table S3).

**Fig. 2:**
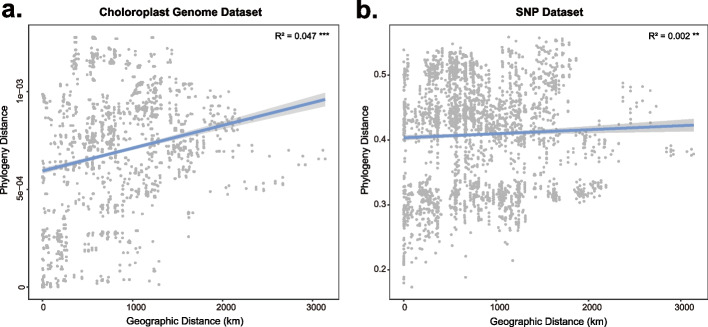
Correlation between the phylogenetic distance of the chloroplast-based or nuclear-based tree and geographical distance

In order to discover the discordance relationships between the chloroplast and nuclear genomes, we compared the two phylogenetic trees (Fig. 1), observing significant incongruence between them. Samples that were presented as closely related (e.g., within-population samples) according to the tree based on the chloroplast genomes did not form a branch in the tree based on the nuclear genomes, suggesting independent evolution in the chloroplast and nuclear genomes. For example, Clade II in the chloroplast genome tree, which only contained the SXGX population, was more highly diverged in the nuclear genome tree. The phylogenetic distance of *Q. aliena* accessions in the chloroplast tree was corrected with geographical distance (*R*^2^ = 0.047) (Fig. 2a), indicating that the chloroplast tree exhibited consistency with the geographical distribution while showing inaccurate genomic divergence history. The nuclear tree also showed correlation with the geographical distribution, but the correlation value was extremely low (*R*^2^ = 0.002) (Fig. 2b).

### Population genetic divergence and genetic diversity

The population structure analysis was conducted using admixture and PCA methods based on the chloroplast genome and nuclear SNPs datasets. A PCA based on the entire chloroplast genome sequences showed four significant principal components (PCs; Figure S3), which was consistent with the phylogenetic relationships. The first two PCs explained > 32.67% of the total variance. However, the population structure assignment had some discordance with the phylogenetic relationships (Fig. 1a): Clade IV did not form an independent genetic structure but had two genetic backgrounds.

**Fig. 3:**
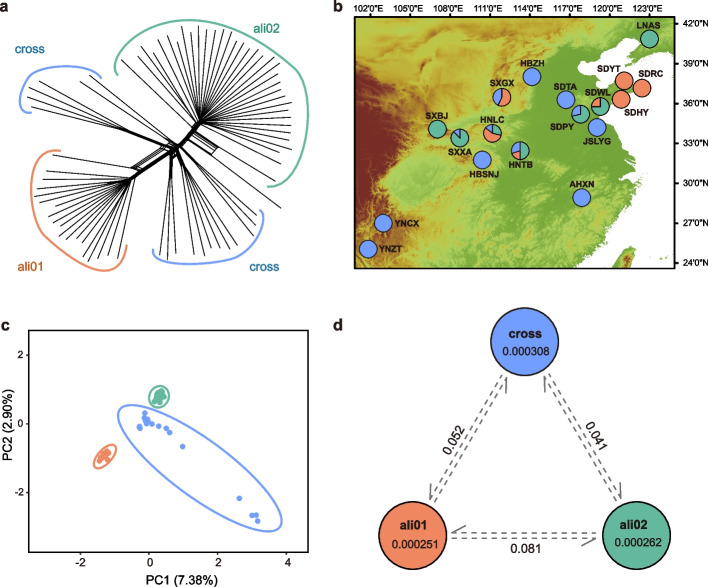
Geographical distribution, genetic divergence, and genetic diversity patterns of *Quercus aliena.* **a** Neighbor-Net network inferred from nuclear SNPs, samples with both major components of genetic structure less than 0.8 were classified as hybrids (the “cross” group). **b** The geographical distribution of 18 populations of *Q. aliena.* Pie charts show the ancestry composition of each population for K = 2 inferred using Admixture. The elevation distribution map in the background was obtained from WorldClim (https://worldclim.org/). **c** Principal component analysis of *Q. aliena* based on nuclear SNPs. The first two principal components explained 7.38% and 2.90% of the total variance, respectively. **d** Genetic differentiation (*F*_*ST*_) among three groups and the nucleotide diversity (π) in each group according to the results of Admixture with K = 2

For the nuclear SNP dataset, the lowest cross validation (CV) error was K = 2. The ali01 group contained 18 accessions from Henan, Shandong, and Shanxi, while the ali02 group included 34 accessions from Henan, Shandong, Shanxi, and Liaoning. Samples with both major components of genetic structure less than 0.8 were classified as hybrids (the “cross” group), which containing 20 accessions, according to the admixture coefficient with K = 2 (Fig. 3b, Table S3). The PCA and Neighbor-Net network analyses confirmed the patterns of genetic differentiation detected by the ADMIXTURE algorithm (Fig. 3a and c). Within the tree groups, population differentiation was higher in the “cross” group (π = 0.000308), which were detected gene flow from the other two groups (Table S4). The genetic divergence showed significant difference between the ali01 and ali02 groups (*F*_*ST*_ = 0.081).

### Population demographic history

**Fig. 4:**
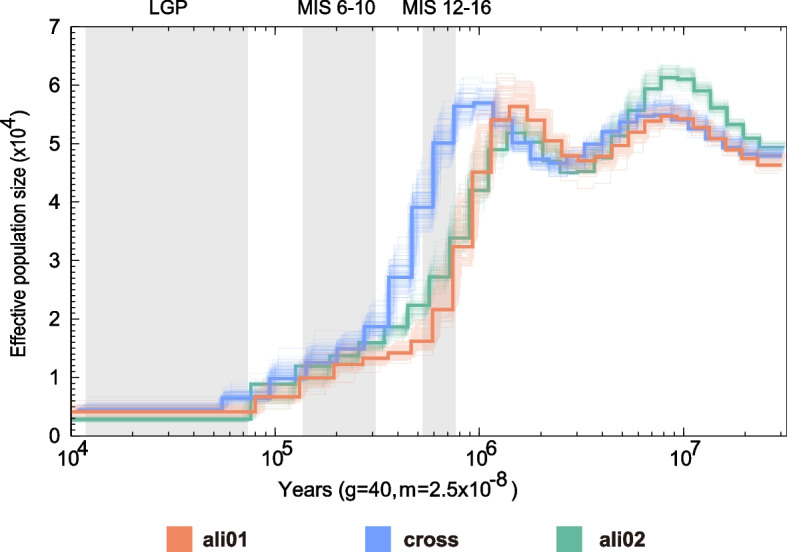
Demographic history analyses. PSMC was used to evaluate the dynamic changes in effective population size (*N*_*e*_) through time. LGP: Last Glacial Period, MIS: Marine Isotope Stage

To examine the evolutionary histories of the three groups of *Q. aliena*, a PSMC analysis was performed to investigate the dynamic changes in the effective population size (*N*_*e*_) over time for each group (Fig. 4). The results showed the three groups had a consistent trend of *N*_*e*_ changes, revealing two periods of population decline. The PSMC showed the first bottleneck for each lineage occurred around 1.0–0.4 Mya, during which there was a sharp decline in *N*_*e*_. From 0.4–0.008 Mya, the *N*_*e*_ of the three groups continuously decreased. The cross group showed a larger contemporary *N*_*e*_ than the other two groups, reflecting the much wider range of distribution in the cross group (Fig. 3b). In the LGP period, *N*_*e*_ did not change significantly in any of the groups.

### Intraspecific variation in genotype–climate association

**Fig. 5:**
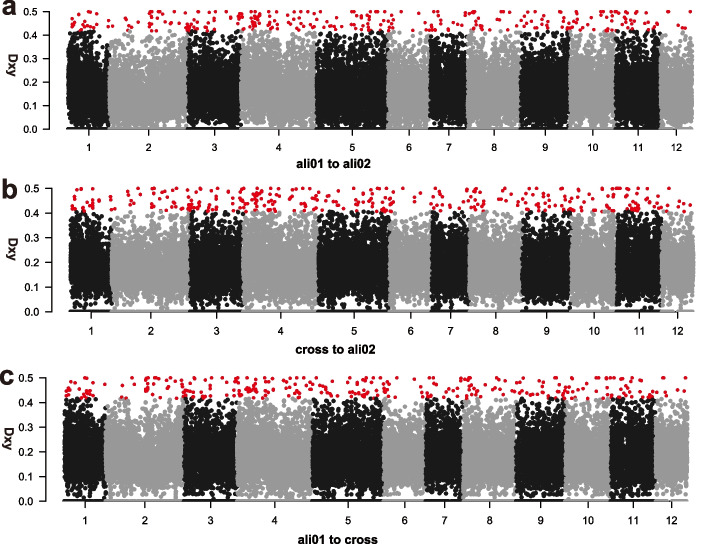
Pairwise absolute genetic divergence (*D*_*XY*_) for pairs of groups along each chromosome of the nuclear genome of *Q. dentata.* The red dashed lines indicate the top 1% of the empirical *D*_*XY*_ distribution

**Fig. 6:**
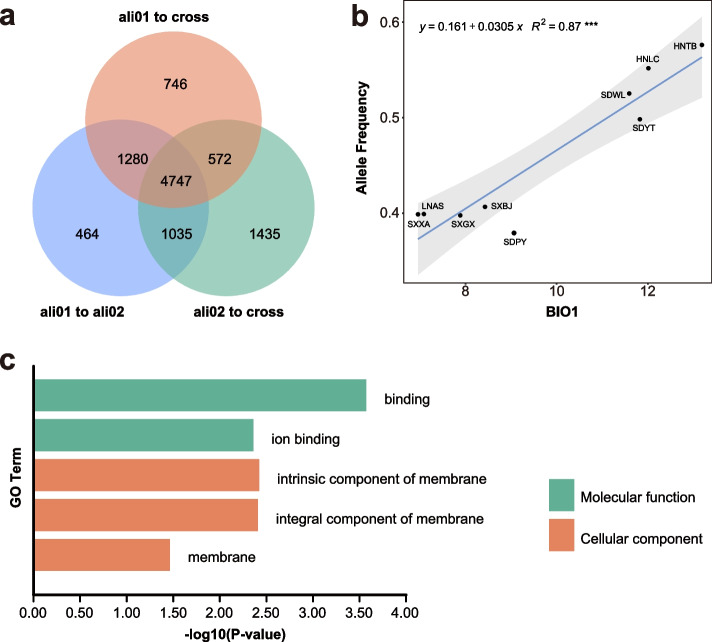
Intraspecific variation in genotype–climate association. **a** Venn diagram depicting overlaps between outlier SNPs detected among the three groups. **b** Relationships between the allele frequency of SNP and BIO1 in *Q. aliena*. Light grey shading indicates the 95% confidence interval of the regression. **c** The GO term or pathway function annotation of highly divergent genes; only GO terms of significantly enriched genes were shown

To investigate the main drivers of genetic differentiation in *Q. aliena* populations, we scanned for heterogeneous genetic variation across the genome using *D*_XY_ (Fig. 5) with 20 kb windows. We detected similar patterns of genetic divergence between the ali01 and ali02 groups, as well as between ali01 and cross, and ali02 and cross. The genomic regions with *D*_XY_ within the top 1% of the empirical *D*_XY_ distribution showed similar patterns across the genome in the three groups (Fig. 5). The intersections of the top 1% windows of the three groups were selected as the highly divergent genomic regions (HDRs) of *Q. aliena* and used in subsequent analyses. Finally, we identified a total of 301 HDRs, which included 4,747 highly divergent SNPs (Fig. 6a), involving 52 genes. GO analysis detected 52 genes related to molecular function and the cellular component process (Fig. 6c).

To explore the key factors contributing to genetic difference between populations, we compared the allele frequencies of the 4,747 highly divergent SNPs with climate variables after remove populations with less than 3 individuals. The positive correlation between allele frequency variation in the SNPs and the annual mean temperature (BIO1) suggest a potential capacity of this species to adapt to changing temperatures (Fig. 6b).

## Discussion

### Chloroplast genome variation in *Quercus aliena*

As chloroplast genomes have a lower substitution rate than nuclear genomes, they are mostly used for evolutionary studies at the species level or higher [20–25]. Chloroplast genomes are less commonly used at the intraspecies level owing to less polymorphism in practice [19, 26, 27]. However, with the advent of NGS sequencing methods, whole chloroplast genomes have been sequenced and used in population studies, such as of the medicinal plants *Arnebia euchroma* and *Arnebia guttata* [28], the endangered species *Ulmus laevis* [29] and *Bretschneidera sinensis* [30], the fruit tree *Ziziphus jujuba* [31], and the ornamental plants *Aquilegia ecalcarata* [15] and *Lagerstroemia indica* [32]. These findings suggest that chloroplast genome sequences contain many SNPs and indels suitable for genetic diversity and phylogeography studies.

This study analyzed the chloroplast genomes of 72 *Q. aliena* accessions, identifying 550 SNPs and 161 indel variations with higher intraspecies variability.. Two hypotheses have been used to explain the heterogeneity rates across different plant groups. The first is that the long divergence times between populations enable more mutations to accumulate. The second is that mutation rates are negatively correlated with generation times, which suggests that long-lived plants may have lower mutation rates than short-lived species. *Q. aliena* is a woody plant with a long generation time, and this habit may explain the lower mutation rate of its chloroplast genome.

Additionally, there is mutation heterogeneity in various areas of the chloroplast genome, such as in “mutation hotspots”, where mutation rates are higher. For example, it is well known that the LSC and SSC regions are less conserved than the IR region. The three identified variable markers (*trnG-trnR-atpA*, *psbM-trnD*, and *trnS-psbZ-trnG*) in the *Q. aliena* chloroplast genome were located in the spacer region in the LSC.

### Cytonuclear discordance in *Quercus aliena* populations

Cytonuclear discordance refers to markedly different topological patterns between nuclear and chloroplast or mitochondrial genomes, and is a common phenomenon in the tree of life [33–35]. Previous studies have shown many cases of cytonuclear discordance at the species level or above. This may be caused by several processes; for example, gene introgression and ancient hybridization may load to different topologies at deeper nodes, such as at the tribe level in the olive plant family [36] and at the subfamily level in Amaranthaceae s.l. [37]. The most frequently invoked mechanism of discordance—incomplete lineage sorting—may be caused by a large effective population size, for example in the *Catalpa* [35] and *Polemonium* [38]. However, it has received less research focus within studies on the cytonuclear discordance among different populations. In this study, the different *Q. aliena* populations exhibited significantly different topologies with significant discordance (Fig. 1).

Sex-biased dispersal may simply be due to cytonuclear discordance and may stand out as the most probable explanation for phylogenetic differences within *Q. aliena*. The chloroplast phylogeny among the individuals of *Q. aliena* showed a consistent geographical pattern (Fig. 2a). It is clear that significant pollen dispersal effectively connects several populations for nuclear DNA, while the chloroplast is largely separate. All *Quercus* species are wind pollinated, and their pollen can be dispersed over several kilometers, whereas their fruits (acorns) are heavy and only partially distributed by scatter-hoarding rodents Sciuridae (squirrels), Covidae (jays), and Picidae (woodpeckers) [39]. The populations of *Q. aliena* were divided into two genetic groups (Fig. 3); during a period of isolation, the two groups accumulated changes in both their nuclear and chloroplast genomes. Due to significant pollen gene flow, the two groups were able to exchange genetic material and create a large hybrid zone (Fig. 3), but due to poor seed dissemination, the chloroplast genomes exchanged little information and produced a dividing line. Irwin [40] argued that in continuous populations without geographical isolation, significant dispersal limitations can easily lead to deep divergence of chloroplast or mitochondrial DNA with nuclear DNA. We therefore believe that sex-biased dispersal-related differences in pollen and seed gene flow rates are the most likely cause of chloroplast and nuclear discordance.

### Genomic diversity, demographic history and climate adaptation of *Quercus aliena*

As an important subtropical and temperate forest tree, *Q. aliena* has important ecological and economic value, and it is therefore essential to reveal the population dynamics, genetic diversity, and genetic structure of the species. In this study, *Q. aliena* was revealed to have high genetic diversity. The reasons for this may be: 1) the high genetic diversity is related to the evolutionary history of the plant: *Q. aliena* is the most widely distributed species in the genus *Quercus*, and the ancestral populations had rich genetic variation, with frequent gene flow throughout the species’ evolutionary history (Fig. 3); 2) *Quercus* is a perennial woody plant that is wind-pollinated, with heterogeneous pollination. The field survey found that *Q. aliena*'s current populations are still wild, with relatively intact population composition and low anthropogenic disturbance; moreover, plants of all ages occur in adequate numbers, thus avoiding adverse effects on the species’ genetic diversity; 3) genetic diversity is positively correlated with the geographical distribution range, and species with wide distribution ranges have high genetic diversity: *Q. aliena* is the most widely distributed species in the genus *Quercus*, with a wide variety of habitats, which provides conditions for *Quercus* to accumulate rich genetic diversity; and 4) the populations of *Q. aliena* can been divided into two genetic groups, and frequent hybridization gene flow between these two genetic groups has led to a hybrid zone and formation of multiple hybrid genotypes (Fig. 3).

The population demographic history analyses showed the effective population size sharply declined about 1 Mya (Fig. 4), which is consistent with analyses of other temperate forests trees such as Asian butternut (Juglans section Cardiocaryon) [33] and Populus species [41]. During the Quaternary Period, global climatic changes influenced the demography and distribution of most plant species. Many species experienced severe habitat constriction or loss during glacial periods [42]. As a result, these species either became extinct or were forced to migrate, where they survived in glacial refugia and adapted to the new environment. For instance, the “founder effect” during postglacial expansions or genetic bottlenecks in glacial refugia may have caused a decline in genetic diversity and the Ne [43]. In Eastern China, there were three major glacial episodes throughout the Quaternary period: the Poyang Glaciation (PG, 0.9–1.2 Mya), the Da Gu Glaciation (DGG, 0.68–0.8 Mya), and the Lushan Glaciation (DG, 0.24–0.37 Mya) [44]. Our PSMC results showed the genetic bottlenecks of *Q. aliena* mainly occurred in the Poyang Glaciation period (Fig. 4). Genotype-climate analysis supported temperatures were the key environmental factors contributing to genetic divergence in *Q. aliena* (Fig. 6), on the other hand, *Q. aliena* retained higher genetic diversity in warmer regions, which also represented higher adaptability to warmer environments at the genetic level and explained its rapid re-expansion during interglacial (120-11Kya).. Furthermore, it is possible that the geographical separation of populations in glacial refugia sped up population differentiation and, in some instances, brought about allopatric speciation [45]. There was significant genetic differentiation between *Q. aliena* populations (Figs. 1 and 3). The uplift of the mountains in northern China may have contributed to the genetic divergence of *Q. aliena*, such as Taihang mountains, which had been uplifted multiple times from 1.7 Ma [46].Material and methods.

### Samples, climate variables, and whole-genome resequencing

We collected 72 individuals of *Q. aliena* across 18 population and one sample of *Q. serrata* from filed in China, the voucher specimens were deposited at the Herbarium of Shandong Provincial Center of Forest and Grass Germplasm Resources and the sample details were shown in Table S1. Biao Han identified all samples. Fresh leaves were harvested from mature trees that were at least 50 m apart in each population. Climate variables for the different populations were obtained from WorldClim (https://worldclim.org/) at a 2.5-min resolution.

We used a modified CTAB method [47] to extract genomic DNA from the leaves dried with silica gel. DNA concentration was measured with a QUBIT 2.0 fluorometer (Invitrogen). We used ultrasonic fragmentation to break down the total DNA to 350 bp and constructed paired-end sequencing libraries with an insert size of 350 bp following the Illumina manufacturer’s instructions. All the libraries were performed on the Illumina Hiseq Xten sequencing system at Novogene (Tianjin, China), and each sample generated about 25 Gb of data, with a target coverage of 30 × .

We used Trimmomatic version 0.39 [48] to process the raw sequence reads, removing adapter sequences and bases with quality lower than Q20 (Phred quality score < 20) from both ends.. After trimming, we removed readings that were less than 50 bp in length.

### Chloroplast genome assembly and variation analysis

To lower computational costs, we split about 4 Gb of clean data to assemble the whole chloroplast genomes of all the *Q. aliena* individuals. We used GetOrganelle [49] with a range of k-mers of 75, 85, 95, and 105 for chloroplast genome assembly. When GetOrganelle failed, we adopted the methods of Dong et al. [36]. Genes of the chloroplast genomes were annotated using Plann [50], and the published genome of *Q. aliena* (GenBank accession number: KP301144) served as the reference sequence. Chloroplot [51] was used to draw the chloroplast genome physical maps of *Q. aliena*.

We aligned the assembled chloroplast genomes of all *Q. aliena* individuals using MAFFT version 7.490 [52] and manually adjusted using Se-Al version 2.0 [53]. To prevent overestimating sequence divergence, alignment problems related to polymeric repeat structures and small inversions were rectified manually.

We used nucleotide diversity and the number of variable and parsimony-informative sites to measure sequence divergence across all the chloroplast genomes. The number of variable and parsimony-informative sites and nucleotide diversity (π) were calculated using MEGA 7.0 [54] and DnaSP v6 [55].

### SNP calling

At present, there is no complete nuclear genome of *Q. aliena* available. Based on phylogenetic relationships of *Quercus*, *Q. aliena* and *Q. dentata* form a clade, and have a relatively close genetic relationship. Therefore, we used the nuclear genome of *Q. dentata* as the reference genome [56] to call the SNPs. We aligned the high-quality reads using the program BWA version 0.7.17 [57] with default settings. The results of the alignments were sorted using SAMtools version 1.3.1 and filtered the PCR duplicates using Picard tools version 1.92 (http://broadinstitute.github.io/picard/). We used GATK version 4.2.0.0 [58] to call SNPs. We removed the low-quality SNPs using strict criteria as follows: SNPs with more than two alleles in the dataset; sites with coverage below 2; sites with sample coverage < 90%. Finally, we kept 1,149,758 high-quality SNPs, called from 72 individuals across 18 populations, for subsequent analyses.

### Phylogenetic and population structure analyses

The whole chloroplast genomes and the nuclear SNP datasets were used to investigate genetic relationships among the accessions.. A phylogenetic tree based on the whole chloroplast genome of *Q. aliena* was reconstructed using a maximum likelihood (ML) method in RAxML-NG [59], with *Quercus serrata* used as the outgroup. We used ModelFinder [60] based on Bayesian information criteria to find the best-fitting model for nucleotide sequence evolution for the ML analysis.

We used both concatenation- and coalescent-based methods to estimate the nuclear phylogeny. For the concatenation-based method, we used IQ-TREE version 2 [61] to construct the ML tree and UFBoot method was used to computed branch support values. The species tree was summarized from all 100 bootstrap replicates.SplitsTree3 was used to perform a Neighbor-Net network, [62], which generated an unrooted network with which to explore complex evolutionary processes such as gene flow, introgression, and/or hybridization.

We used admixture analysis and principal component analysis (PCA) based on both the chloroplast genome (–haploid “*” were set) and nuclear SNP datasets to infer population structure and population assignments. First, Admixture version 1.3.0 was run with predefined clusters (K = 1–10) and conducted ten runs for each K value. We determined the best K with the lowest cross-validation error methods. We also reported the other number of genetic clusters that made biological sense, and detected the gene flow between different groups from Admixture of SNP dataset by Dsuite [63]. Then, PLINK was used for PCA analysis of the SNP dataset [64].

### Population demography

We used a pairwise sequentially Markovian coalescent (PSMC) model to infer dynamic fluctuations in the effective population size (*N*_*e*_) of the *Q. aliena* populations through time [65]. We used the BWA/SAMtools pipeline to obtain the consensus sequences for each sample while masking the bases with exceptionally low and high coverage. We then carried out PSMC with the settings “-N35 -t15 -r4 -p 4 + 25*2 + 4 + 6.” A generation time of 40 years and an assumed mutation rate of 2.5 × 10^–8^ per site per generation were used to scale the results. After combining all the resulting files, we used “PSMC plot.pl” from the PSMC package to create the graphs.

### Genome-wide genetic diversity and differentiation

We analyzed the genomic diversity and differentiation at the genome-wide level to identify the genetic divergence among the different population groups of *Q.aliena*. Genome-wide differentiation between the population groups was measured using pairwise nucleotide difference (*D*_*XY*_) in VCFtools with 20 kb non-overlapping sliding windows. The top 1% of windows with the highest *D*_*XY*_ values were regard as the highly divergent genomic regions (HDRs) of the different groups; SNPs as well as related genes distributed in HDRs were selected as the highly divergent sites.

To determine whether these highly divergent genes were enriched with any functional classes of genes, functional enrichment analysis using Gene Ontology (GO) was performed in TBtools with Fisher’s exact test used to assess significance [66]. Fisher's exact test P values were corrected using the false discovery rate and GO terms with a corrected *P* < 0.05 were considered to be significantly enriched.

### Supplementary Information

**Additional file 1: Figure S1.** Distribution range of Q. aliena in China, based on specimens from NSII (http://www.nsii.org.cn/).

**Additional file 2: Figure S2.** Nucleotide diversity of the Q. aliena chloroplast genomes. The window size was 800 bp and the step size was 100 bp.

**Additional file 3: Figure S3.** Principal component analysis (PCA) of Q. aliena based on chloroplast genomes. The first two principal components (PC1 and PC2) explained 18.84% and 13.83% of the total variance, respectively.

**Additional file 4: Figure S4.** The geographical distribution of 18 populations of Q. aliena. Pie charts show the ancestry composition of each population for K = 3 based on Admixture results of chloroplast genome. The elevation distribution map in the background was obtained from WorldClim (https://worldclim.org/).

**Additional file 5: Table S1.** Sample information and the size of the chloroplast genome.

**Additional file 6: Table S2.** Admixture results from chloroplast genome dataset

**Additional file 7: Table S3. **Admixture results from SNP dataset.

**Additional file 8: Table S4. ** ABBA-BABA analysis from Dsuite.

## Data Availability

The sequenced raw data are deposited in the SRA database with the accession number of PRJNA985072.
