# Supplementary material for: Genomic divergence and demographic history of Quercus aliena populations
Source: BMC Plant Biol. 2024 Jan 9;24:39. doi: 10.1186/s12870-023-04623-y (PMC10775429; doi:10.1186/s12870-023-04623-y)
Supplement: Supplementary file 1 — Additional file 1: Figure S1. Distribution range of Q. aliena in China, based on specimens from NSII (http://www.nsii.org.cn/). [file 12870_2023_4623_MOESM1_ESM.pdf]

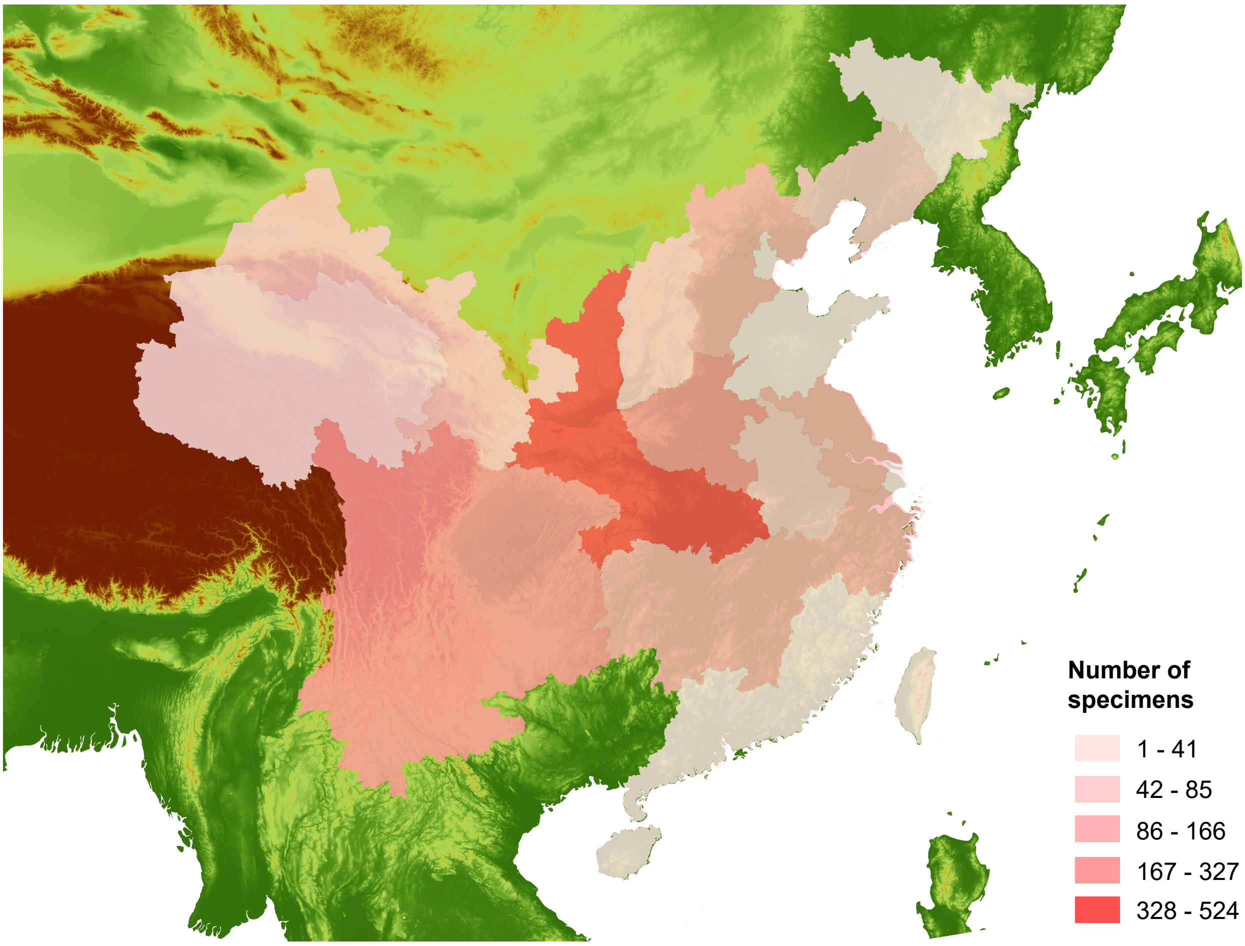

Figure S1. Distribution range of *Q. aliena* in China, based on specimens from NSII (http://www.nsii.org.cn/)
