# Supplementary material for: Genomic divergence and demographic history of Quercus aliena populations
Source: BMC Plant Biol. 2024 Jan 9;24:39. doi: 10.1186/s12870-023-04623-y (PMC10775429; doi:10.1186/s12870-023-04623-y)
Supplement: Supplementary file 2 — Additional file 2: Figure S2. Nucleotide diversity of the Q. aliena chloroplast genomes. The window size was 800 bp and the step size was 100 bp. [file 12870_2023_4623_MOESM2_ESM.pdf]

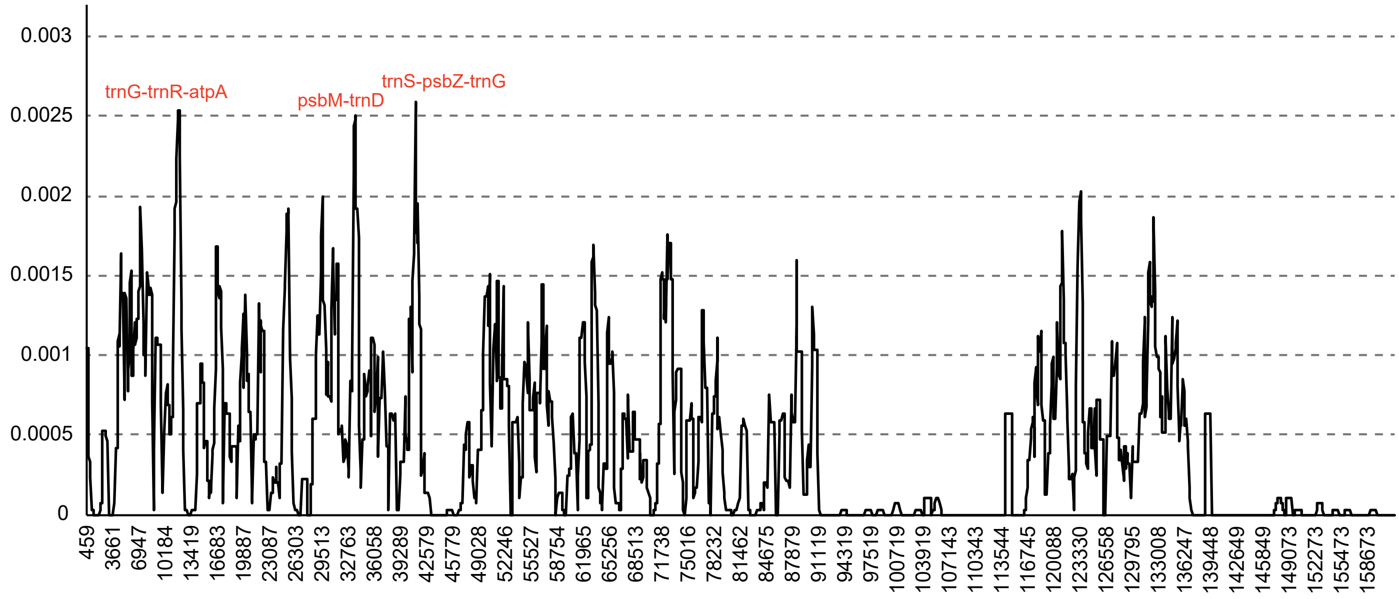

Figure S2. Nucleotide diversity of the *Q. aliena* chloroplast genomes. The window size was 800 bp and the step size was 100 bp.
