# Supplementary material for: Genomic divergence and demographic history of Quercus aliena populations
Source: BMC Plant Biol. 2024 Jan 9;24:39. doi: 10.1186/s12870-023-04623-y (PMC10775429; doi:10.1186/s12870-023-04623-y)
Supplement: Supplementary file 3 — Additional file 3: Figure S3. Principal component analysis (PCA) of Q. aliena based on chloroplast genomes. The first two principal components (PC1 and PC2) explained 18.84% and 13.83% of the total variance, respectively. [file 12870_2023_4623_MOESM3_ESM.pdf]

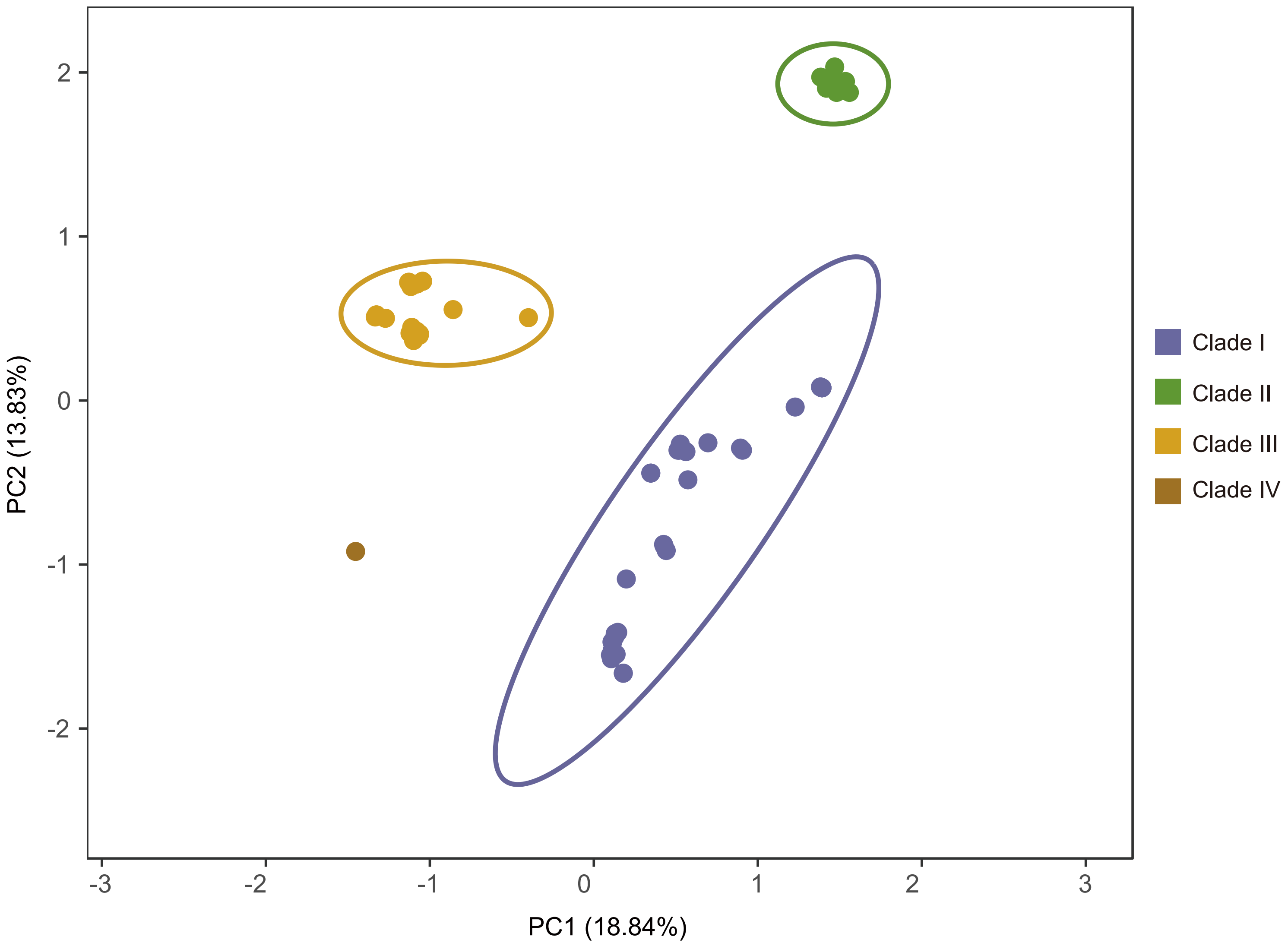

Figure S3. Principal component analysis (PCA) of *Q. aliena* based on chloroplast genomes. The first two principal components (PC1 and PC2) explained 18.84% and 13.83% of the total variance, respectively.
