# Supplementary material for: Genomic divergence and demographic history of Quercus aliena populations
Source: BMC Plant Biol. 2024 Jan 9;24:39. doi: 10.1186/s12870-023-04623-y (PMC10775429; doi:10.1186/s12870-023-04623-y)
Supplement: Supplementary file 4 — Additional file 4: Figure S4. The geographical distribution of 18 populations of Q. aliena. Pie charts show the ancestry composition of each population for K = 3 based on Admixture results of chloroplast genome. The elevation distribution map in the background was obtained from WorldClim (https://worldclim.org/). [file 12870_2023_4623_MOESM4_ESM.pdf]

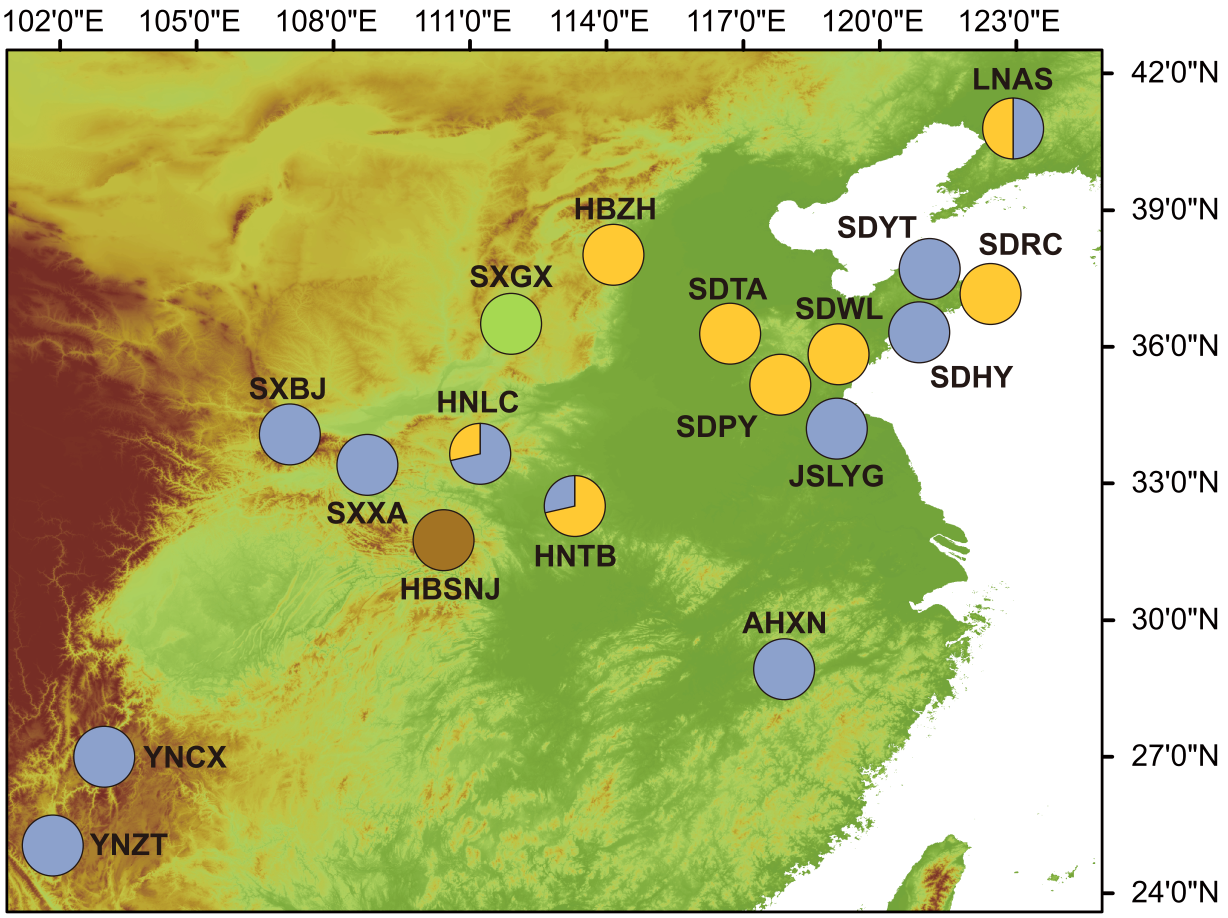

Figure S4. The geographical distribution of 18 populations of *Q. aliena*. Pie charts show the ancestry composition of each population for K = 3 based on Admixture results of chloroplast genome. The elevation distribution map in the background was obtained from WorldClim (https://worldclim.org/)
